# Obituary

**Published:** 1905-05-15

**Authors:** 


### OBITUARY.

Dr. Waye was borne in Central New York, in 1826, where he resided until he reached his majority. He began the study of dentistry in that State, and continued to practice there for a short time before settling in Ohio. In 1858, Dr. Waye opened an office in Sandusky and continued to practice there until 1891, when he retired. From that date until his decease, his health gradually failed. He was a member of the Ohio State Dental Association, and of the Northern Ohio Dental Association, of which latter he was the president at one time. He was quite a prolific writer on dental subjects, contributing many able articles to the different journals. The honorary degree of Doctor of Dental Surgery was conferred on him by the Ohio Dental College in 1874.

He was a man of strong mental vigor, and was always ready to lend sympathy and assistance to the younger members of his profession. He died April 2nd, 1905, at the ripe age of seventy-nine years, leaving a record of sterling integrity and upright character, having enjoyed the confidence and respect of all who knew him, especially members of his profession.

